# Shared early processing of distinct tactile features

**DOI:** 10.1016/j.isci.2025.114485

**Published:** 2025-12-18

**Authors:** Michaela Jeschke, Elena Azañón, Knut Drewing

**Affiliations:** 1 Department of Experimental Psychology, Justus-Liebig University, 35394 Giessen, Germany; 2 Institute of Psychology, Otto von Guericke University Magdeburg, 39106 Magdeburg, Germany; 3 Leibniz Institute for Neurobiology, 39118 Magdeburg, Germany; 4 Department of Neurology, Otto von Guericke University Magdeburg, 39120 Magdeburg, Germany; 5 Center for Behavioral Brain Sciences, 39106 Magdeburg, Germany

**Keywords:** health sciences

## Abstract

The extent to which spatial tactile properties share neural pathways remains unclear, yet it is the key to understanding how the brain constructs coherent object representations from distributed spatial inputs. One basic spatial property is the perceived tactile distance between two simultaneous touches on the skin. It exhibits adaptation aftereffects: when body areas are repeatedly touched at two points, subsequently presented smaller distances are perceived as smaller than on unadapted areas. We investigated whether tactile distance adaptation influences the perception of other spatial properties, macro-scale roughness and curvature, indicating shared neural mechanisms. In experiment 1, adapting the skin to a fixed tactile distance reduced perceived roughness of subsequent gratings with smaller groove widths, as assessed through passive touch at the finger pad. This aftereffect likely originates from early cortical processing, as it is orientation-specific and independent of peripheral receptor desensitization. Experiment 2 demonstrated that curvature perception increases after adaptation to a two-point distance larger than the curve, suggesting overlap in processing pathways. Experiment 3 further supported early processing involvement, as the distance-to-roughness aftereffect did not transfer to adjacent skin regions of the same finger. Experiment 4 revealed bidirectional aftereffects: roughness adaptation also influenced distance perception. However, within-property aftereffects were stronger than cross-property effects. By revealing the existence of cross-property adaptation aftereffects with low-level characteristics, our findings provide evidence that tactile distance, roughness, and curvature share early somatosensory processing. This suggests that spatially defined properties undergo a common initial processing stage, sharing initial steps rather than existing in a hierarchical processing arrangement.

## Highlights

• Tactile distance, roughness, and curvature show overlaps in their somatosensory processing
• Adapting to one tactile property produced measurable aftereffects in another
• Findings likely indicate a shared early cortical basis for spatial tactile properties

## Graphical abstract

**Figure:**
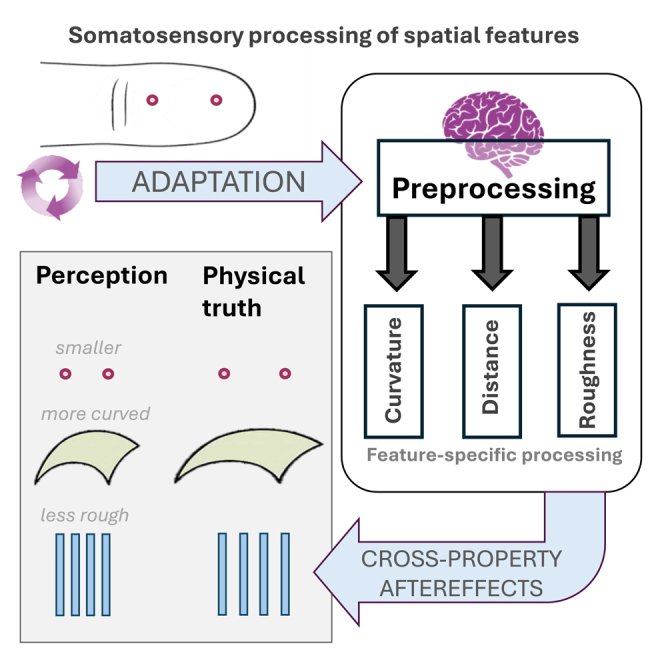

## Introduction

Humans continuously extract tactile information to perceive objects and materials, whether through active exploration or via passive touch. Here, we investigate basic mechanisms underlying somatosensory processing during passive touch, focusing on how the brain combines spatial representations from tactile inputs. Specifically, we examine tactile distance, i.e., the perceived distance between two spatially separated points on the skin, and its relationship with surface roughness and curvature perception. While these properties are often studied in isolation,^1^^,^^2^^,^^3^^,^^4^^,^^5^^,^^6^ it remains unclear whether they rely on distinct or overlapping processing mechanisms. Addressing this question can reveal whether the brain generalizes spatial coding strategies across domains or maintains specialized processing streams for related but functionally distinct types of spatial information.

The perception of tactile distance has been found to be a basic somatosensory process^7^ that is strongly modulated by low-level influences like receptor density, anisotropies in the shape of tactile receptive fields (RFs), and resulting differences in cortical magnification.^1^^,^^8^ The computation of the distance between isolated points may be related to the encoding of critical properties in object discrimination, such as roughness and curvature: while tactile distance likely reflects fundamental spatial organization, both roughness and curvature perception also rely heavily on the integration of spatial cues,^9^^,^^10^ making computational similarities between them plausible. In the current study, we examined whether certain mechanisms underlying the processing of spatial distance overlap with those involved in roughness and curvature, in order to infer the cortical level at which these interactions occur, and to determine whether one property serves as a foundational scaffold for processing the other properties. Understanding processing overlap and distinguishing between these two possibilities, i.e., common early processing versus sequential hierarchical integration, is essential for understanding how and when discrete tactile cues are combined to support object perception, as features like separation, surface variation, and curvature are central to recognizing material properties and object identity.

To address these questions, we leveraged tactile adaptation aftereffects as a means to examine cross-property interactions. Adaptation aftereffects, i.e., the change in perception of stimuli after prolonged exposure to another stimulus, are a widely used psychophysical tool,^11^ as they allow researchers to infer how different stimulus dimensions are processed e.g., by populations of selective neurons.^12^ All properties in the scope of this study have been previously shown to be subject to aftereffects;^7^^,^^13^^,^^14^ Calzolari and colleagues demonstrated, for instance, that after adaptation to a tactile distance, participants perceive subsequent smaller distances as smaller than on unadapted skin areas.^7^ Note that in both Calzolari et al. and the present study, we focus on the computation of the abstract property of distance (e.g., 2 cm) between two points, independent of their specific location on the skin. This is achieved by repeatedly presenting two isolated, simultaneous points across different skin areas on the finger pad or hand. While localized RF-based processing is necessary for computing distance, our approach ensures that this computation is not tied to two particular RFs. Instead, it allows for a more generalized perception of distance that transcends specific spatial arrangements. To study the interplay between the different properties, we used a modified approach and investigated whether adaptation to one property can affect perception of the other, producing a corresponding aftereffect. Such cross-property aftereffects have been only rarely employed and predominantly in the context of visual aftereffects.^15^^,^^16^^,^^17^^,^^18^ To our knowledge, only one study employed a similar approach for touch, demonstrating that adaptation to high frequency vibrations (>100 Hz) impaired perception of fine textures but left perception of coarse textures mostly unaffected.^19^

**Figure 1:**
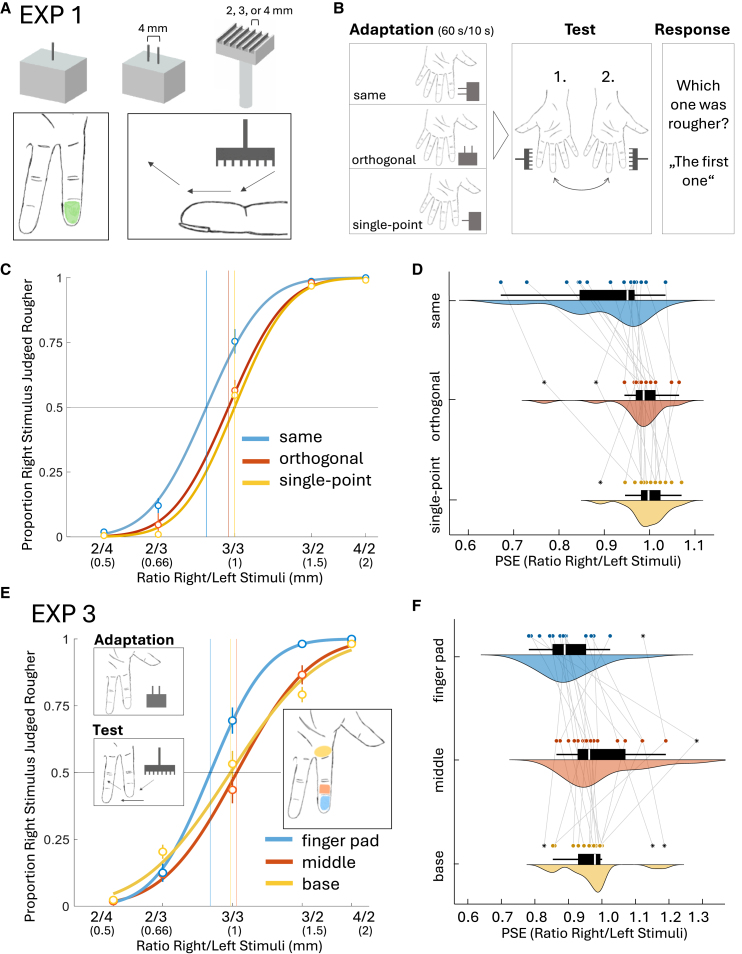
Procedure and results of experiments 1 and 3 (A) Single-point adaptation stimulus, two-point adaptation stimulus, one example of the three test gratings (first row); application area of adaptation at left-hand index finger pad, application procedure of test grating on both index finger pads (second row). (B) Experimental procedure. (C) Psychometric functions for the averaged proportions of the same orientation, orthogonal orientation and single-point adaptation conditions (*N* = 18). Every data point shows the proportion of trials for which participants reported the test stimulus presented to the right index finger as rougher than the stimulus presented to the left (adapted) index finger for each RIF/LIF stimulus ratio. Note that participant’s actual response was whether the first or the second applied stimuli was rougher. Psychometric functions are cumulative Gaussian functions. Error bars represent the SEM. Vertical lines represent mean PSEs of each condition. (D) Combined boxplot, density plot and scatterplot for same orientation, orthogonal orientation and single-point adaptation conditions. Connecting lines indicate data points belonging to the same participant. Boxplot: white line within black box displays median value. Black box spans from Q1 (25th percentile) to Q3 (75th percentile), representing the interquartile range (IQR), containing the middle 50% of the data. Whiskers extend from the box to the smallest and largest values within 1.5 × Interquartile range below Q1 and above Q3, respectively. Points outside this range are considered outliers. (E) Averaged psychometric functions for finger pad, middle phalanx and finger base test area conditions in experiment 3 (*N* = 18), where the effect of adaptation to distance on the left index finger pad was tested on three adjacent areas (finger pad, middle phalanx, and finger base. (F) Combined boxplot, density plot and scatterplot for finger pad, middle phalanx and finger base test area conditions.

We expected that adaptation to a specific tactile distance, coded through two isolated points on the skin, should influence the perception of macro-scale roughness: the Duplex theory of texture perception^20^^,^^21^ postulates that fine textures rely on a temporal-vibratory code, driven by the interaction between the finger and the surface, producing vibrations that are processed by fast and rapidly adapting mechanoreceptors. In contrast, the perception of coarse textures such as those used in this study depends on a spatial code, which is based on the variability in skin deformation.^2^^,^^19^^,^^22^ Perceived roughness of coarse textures is mostly affected by changing inter-element spacing, element width and spatial frequency, with roughness perception increasing as the separation between elements becomes larger.^23^^,^^24^^,^^25^ Hence, we expect that adaptation to a certain tactile distance affects how subsequent groove-ridge gratings are perceived (Figure 1B). Specifically, if gratings with substantially smaller groove widths than an adapted distance are perceived as less rough on the adapted finger, this would provide a striking causal demonstration that spatial coding is fundamental to roughness perception. We tested this in the first experiment of our study (experiment 1).

**Figure 2:**
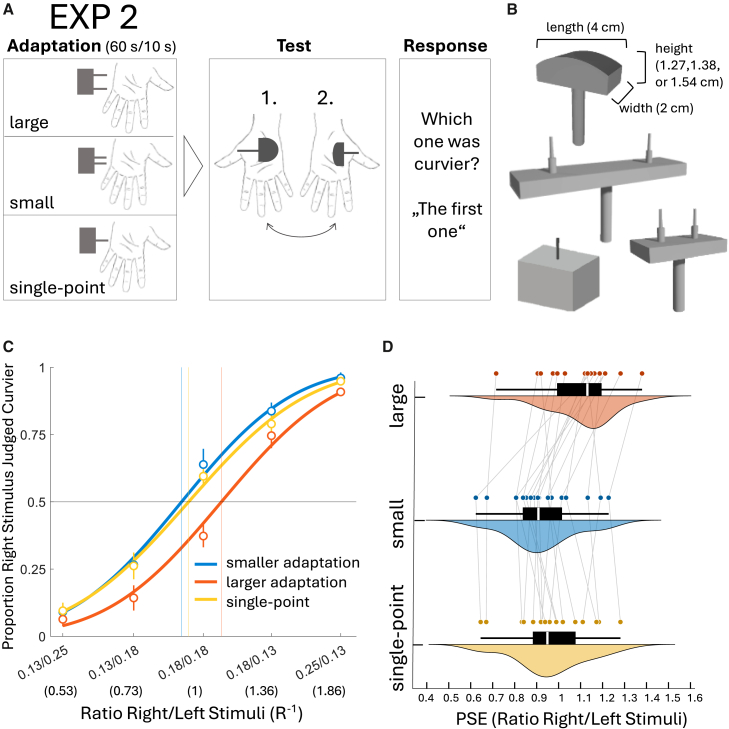
Procedure and results of experiment 2 (A) Experimental procedure, application area of the adaptation stimuli is always at the left-hand palm. (B) Haptic stimuli: one example of the three curvature test stimuli; large two-point distance adaptation stimulus (distance, 4.5 cm), single point adaptation stimulus, and small two-point distance adaptation stimulus (distance, 1.5 cm). (C) Psychometric functions for the averaged proportions of the small adaptation, large adaptation, and single-point adaptation conditions (*N* = 18). Every data point shows the proportion of trials for which participants reported the test stimulus presented to the right hand palm as more curved than the stimulus presented to the left (adapted) hand palm for each RH/LH stimulus ratio. Note, that participant’s actual response was whether the first or the second applied stimuli was more curved. Psychometric functions are cumulative Gaussian functions. Error bars represent the SEM. Vertical lines represent mean PSEs of each condition. (D) Combined boxplot, density plot and scatterplot for small adaptation, large adaptation, and single-point adaptation conditions.

Building on the possibility of shared mechanisms for complex object perception, we investigated in experiment 2 whether adaptation to tactile distance also induces cross-property aftereffects in curvature perception.^4^ To test this, we used convex curved strips (Figure 2B) with constant contact lengths^9^ but differing heights, resulting in varying curvature values. We hypothesized that adaptation to a distance that is notably larger than the length of the subsequently applied curve’s indentation area would lead to an underestimation of the length of the indentation area. With the base-to-peak height staying constant, the gradient of the curve might consequently be perceived as steeper, i.e., perceived as more curved.^5^ Vice versa, when adapting to a notably smaller length, the opposite should happen, i.e., subsequently presented curves would be perceived as less curved. Contrasting effects of large versus small adaptation, i.e., a cross-property aftereffect from tactile distance to curvature, would highlight the importance of distance processing not only for roughness, but for the computation of properties that involve spatial processing in general.

We further aimed to infer the cortical level of processing where such interactions occur. Aftereffects are highly informative regarding the cortical functioning underlying the perception of properties: while low-level aftereffects stem from earlier cortical processing and thus show strong selectivity for stimulus characteristics such as orientation or location,^26^^,^^27^ higher-level aftereffects putatively arise from later cortical processing and can generalize across orientation, location, or stimulus size.^28^^,^^29^ The tactile distance aftereffect has been shown to exhibit typical low-level characteristics, namely orientation- and region-specificity^7^; investigations on roughness and curvature aftereffects, however, so far provided either none or mixed behavioral evidence on their cortical origins.^13^^,^^14^^,^^20^^,^^30^ Here, we assessed a potential orientation-specificity of the cross-property aftereffect from tactile distance to roughness perception in experiment 1. Specifically, we tested whether the cross-property aftereffect only occurred when the application axes of adaptation stimuli and test gratings were aligned, i.e., when the two-point adapter and test gratings both were applied along the length of the finger pad (Figure 1B), and not when the two-point adapter was rotated orthogonally to the test grating (Figure 1B). Such orientation-specificity would suggest that the cross-property aftereffect arises at early stages of somatosensory processing, likely within primary somatosensory cortex (SI).^31^^,^^32^ Additionally, no transfer should occur when the adapter consisted of a single tactile point instead of two (Figure 1B), applied on random locations across the finger pad, serving as a control for desensitization of peripheral mechanoreceptors and reemphasizing cortical involvement. We extended findings on the potential cortical processing level by assessing location-specificity with a third experiment (experiment 3), testing whether the aftereffect spreads across adjacent skin regions of the same finger. Further, we applied both adapter and test stimulus across the width (Figure 1E) instead of along the length of the finger pad as we did in experiment 1, reinsuring that the aftereffect occurs independently of orientation as long as application axes are aligned.

Finally, to assess whether cross-property aftereffects reflect a common preprocessing stage or whether one property acts as an initial processing scaffold for the other, we compared them to their respective within-property aftereffects. Comparisons of aftereffects comprised distance adaptation to roughness perception; the reversed paradigm, i.e., roughness to distance; and the two congruent variants, i.e., roughness to roughness and distance to distance. If adapting to the same property produced for both properties a stronger aftereffect than cross-property adaptation, this would suggest early common non-hierarchical processing underlying the observed interactions: while congruent adaptation conveys all cues relevant for property computation, incongruent adaptation includes only part of them, thus, resulting in weaker aftereffects. This would be the case for both roughness and distance. Alternatively, if one property were located upstream in the somatosensory pathway as a first step in the processing hierarchy, the difference between congruent and incongruent adaptation should be substantially smaller for that upstream property compared to the other. This is because whenever the downstream property is stimulated, the upstream property is also engaged as input, and thus, becomes strongly adapted regardless of congruent or incongruent conditions.

In summary, our results demonstrate cross-property adaptation aftereffects from tactile distance to roughness and curvature which are not due to peripheral desensitization, indicating that tactile distance shares common cortical processing with these properties. We investigated the character of these interactions in more detail for distance and roughness specifically and revealed that the aftereffects are bidirectional, exhibit low-level characteristics such as orientation- and location-specificity, and are weaker than the respective within-category aftereffects. Tactile distance and macro-scale roughness hence appear to undergo a similar early stage of somatosensory processing, likely reflecting shared early-stage mechanisms instead of a hierarchical processing arrangement.

## Results

### Experiment 1

In the first experiment we investigated the existence of cross-property aftereffects from tactile distance to roughness perception to explore a potential processing overlap of the two properties. If adapting to one would affect the perception of the other, this would suggest communalities in the somatosensory processing of the two properties. On each trial, participants’ left index finger pad was adapted to one of three stimuli: a 4 mm distance applied via two simultaneous touches oriented along the length of the finger (i.e., proximodistal; same orientation as the upcoming grating), a 4 mm distance oriented across the finger (mediolateral; orthogonal rotation), or a single-point stimulus serving as control for desensitization (see procedure, Figure 1B). A two-interval forced choice (2IFC) discrimination task followed where gratings were applied on the participants adapted and unadapted homologous finger pad, moved along the length of the finger (Figure 1A), with groove distances of 2, 3, or 4 mm. Participants then had to judge whether the first or the second stimulus was rougher.

From the individual proportion of trials in which participants judged the right index finger stimulus to be rougher, we determined the point of subjectively equal roughness (PSE) in the adapted as compared to the unadapted skin (Figures 1C and 1D). We performed a Friedman test to assess the effect of adaptation mode (three levels: same orientation, orthogonal rotation, and single-point) on individual average log PSEs. This non-parametric test, appropriate for comparing related conditions, was chosen because the data violated the assumption of normality (Shapiro-Wilk test, *p* < 0.001). Results showed that cross-property aftereffects varied depending on the adaptation mode, as indicated by a significant main effect of the within-participant factor adaptation mode on individual log PSEs (Friedman test, χ^2^[2] = 19.51, *p* < 0.001, *W* = 0.54). Specifically, adaptation to a two-point stimulus applied on the finger pad affected the perception of subsequently presented gratings, decreasing the judged roughness on the adapted finger pad when both adapter and gratings had a matching orientation (Wilcoxon-rank tests; median = 0.942, *MAD* = 0.045; *Z* = 3.0, *p* = <0.001 [one sided], *r* [rank biserial correlation] = 0.97, indicating significant deviations of log PSEs from zero). However, no aftereffects occurred when the adapter was rotated orthogonal to the orientation of the test axis (median = 0.982, *MAD* = 0.025; *Z* = 49.0, *p* = 0.12, *r* = 0.43) or when the adapter consisted of single indentations (median = 0.995, *MAD* = 0.018; *Z* = 75.0, *p* = 0.66, *r* = 0.12). Bonferroni-corrected Conover’s post-hoc tests further confirmed significantly lower PSEs when the adapter and test had the same orientation compared to an orthogonal orientation (*t*[34] = 3.15, *p* = 0.010), or to a single-point adapter (*t*[34] = 4.26, *p* = <0.001). No significant difference was found between the orthogonal and single-point conditions (*t*(34) = 1.11, *p* = 0.83). Note that a previous analysis of the data of experiment 1 has been pre-published in a conference paper.^33^ These results provide causal evidence for the spatial coding postulate and suggest communalities between the processing of tactile distance and roughness. In addition, the absence of aftereffects when the adapter was rotated orthogonal to the orientation of the test axis or when it consisted of only single indentations suggests that the observed interactions arise from early cortical processing.

### Experiment 2

In a second experiment, we examined whether tactile distance adaptation induces a cross-property aftereffect on curvature, assessing whether the relationship observed between tactile distance and roughness in the first experiment also applies to curvature. Such a finding would further emphasize the role of distance processing in the broader computation of spatial tactile properties. To investigate this, we adapted participants’ left-hand palm either to a two-point distance stimulus that was smaller than the indentation area of a subsequently presented curvature stimulus (1.5 cm), to a larger two-point stimulus (4.5 cm), or to a control single-point stimulus. Afterward, a 2IFC discrimination task involving curvature stimuli followed, where participants had to report which stimulus they perceived to be curvier (see procedure, Figure 2A). We conducted both adaptation and test phase at the palm instead of the finger for feasibility reasons (see STAR Methods section). We hypothesized that adaptation to a small distance at the palm of the hand is associated with subsequent decreased curvature perception on the adapted hand, while adaptation to a larger distance leads to increased curvature perception; both effects being produced by changes in perceived indentation length after adaptation to a two-point distance, thereby altering the perception of length/height curvature ratios. A control condition using a single-point adapter should again produce no aftereffect. This pattern was largely reflected in the data: a one-way repeated measures ANOVA on individual log PSEs produced a significant main effect of adaptation mode (three levels: small, large, and single-point) (*F*[2, 34)] = 16.26, *p* = <0.001 *η*^2^ = 0.49). Bonferroni-corrected post-hoc tests between conditions revealed a significant difference between small and large adaptation (*t*[17] = 5.46, *p* = <0.001, *d*_*z*_ = 1.04), with both conditions exhibiting significant deviations from a PSE of 1 (i.e., log PSE = 0) in opposite directions. Thus, adaptation to a small distance significantly reduced the perceived curvature of subsequently applied test stimuli (*M* = 0.90, *SD* = 0.18; one sample *t* test (one-sided), *t*[17] = 2.55, *p* = 0.015, *d* = −0.60), whereas adaptation to a large distance increased perceived curvature (*M* = 1.10, *SD* = 0.172; *t*[17] = 1.98, *p* = 0.032 [one-sided], *d* = −0.47).

No conclusive evidence of an aftereffect was observed for the single-point adaptation (*M* = 0.95, *SD* = 0.18; *t*[17] = −1.53 *p* = 0.14, *d* = −0.36). Comparisons with the single-point condition revealed a significant difference between large and single-point adaptation (*t*[17] = 4.17 *p* = <0.001, *d*_*z*_ = 0.80), but not between small and single-point adaptation (*t*[17] = 1.29, *p* = 0.62, *d*_*z*_ = 0.25). While the absence of a significant difference might suggest partially overlapping effects of small and single-point adaptation, this cannot be interpreted as evidence for equivalence, given the directional aftereffect was observed only for the small adapter. In sum, tactile distance adaptation clearly affected subsequent curvature perception, as demonstrated by the contrasting effects of large versus small distance adaptation.

### Experiment 3

In experiment 3, we aimed to further test our inference regarding the sensory processing level at which the observed interactions between tactile distance and roughness take place. In experiment 1, we observed that the cross-adaptation aftereffect was orientation specific. Here, we assessed the location-specificity of the cross-property aftereffect, testing whether distance adaptation of the finger pad also affects roughness perception on the middle phalanx and finger base (Figure 1E). We expected an aftereffect transfer to the middle phalanx due to cortical spread within SI,^34^ but not as far as to the finger base. Further, contrary to experiment 1, we applied both adapter and test stimulus across the width instead of along the length of the finger pad (Figure 1E), reinsuring that the aftereffect occurs independently of orientation as long as application axis are aligned. Results demonstrate that textures were again perceived as less rough on the finger pad of the adapted finger after adaptation to a two-point distance stimulus, but this aftereffect did not spread across adjacent skin regions of the same finger (Figures 1E and 1F), suggesting that cross-property interactions stem from low-level sensory processing within the primary somatosensory cortex displaying distinct intra-digits separation.^35^ A one-way repeated measures ANOVA revealed a significant main effect of the within-participant factor test area (three levels: finger pad, middle phalanx, and finger base) (*F*[*2*, *34*] = 5.86, *p* = 0.006, *η*^2^ = 0.26). Bonferroni-corrected post-hoc tests between conditions revealed a significant difference between finger pad and middle phalanx (*t*[17] = 3.30, *p* = 0.007, *d*_*z*_ = 1.00) and no difference between middle phalanx and finger base (*t*[17] = 0.87, *p* = >1, *d*_*z*_ = 0.28). However, the difference between finger pad and finger base did not reach significance (*t*[17] = 2.43, *p* = 0.06, *d*_*z*_ = 0.78), although the effect size suggests a substantial difference that may not have been detected due to limited power. In addition, one sample *t* tests against 1 (i.e., log PSE = 0) reemphasized that the transfer of the aftereffect emerged only on the finger pad (*M* = 0.89, *SD* = 0.13, *t*(17) = −3.78, *p* = <0.001 (one-sided), *d* = −0.89), but did not spread to the middle phalanx (*M* = 1.04, *SD* = 0.17; *t*[17] = 0.77, *p* = 0.23 [one-sided], *d* = 0.18), or finger base (*M* = 1.10, *SD* = 0.136; *t*[17] = 0.38, *p* = 0.71, *d* = −0.09).

### Experiment 4

**Figure 3:**
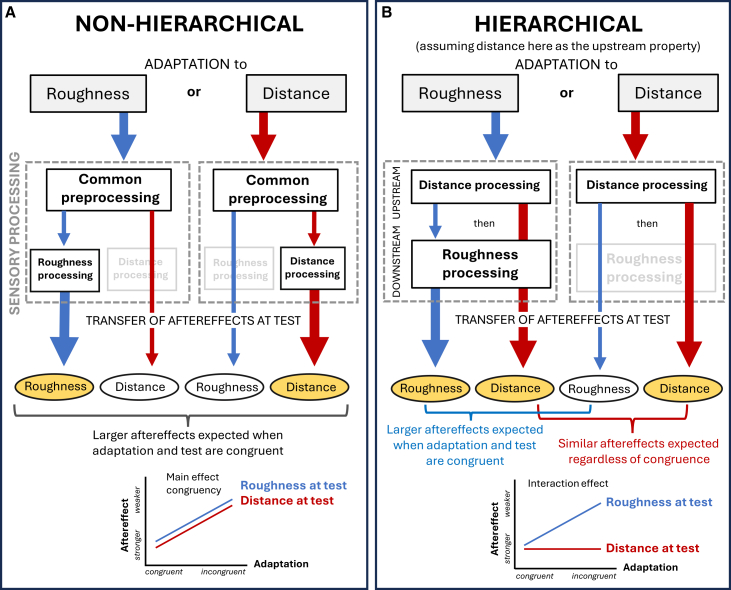
Schematic predictions for within- and cross-property aftereffects under different processing models (A) Non-hierarchical processing; if two perceptual properties share early, overlapping neural computations (“common preprocessing”), cross-property aftereffects are expected in both directions (from roughness to distance and vice-versa) but would be weaker than within-property aftereffects. In both images, the varying thickness of the arrows symbolizes the “adaptation intensity”, which is influenced by the specific processing stage. Below, predictions for the resulting aftereffect strength are depicted in a schematic line-plot. (B) In a hierarchical organization, cross-property aftereffects also occur but show a directional asymmetry. Because processing of a downstream property (here, roughness) builds on representations of the upstream property (here, distance), adaptation to the downstream property automatically engages the upstream stage. Consequently, when testing the upstream property, the difference between congruent and incongruent adaptation will be null or substantially smaller than the difference between congruent and incongruent adaptation when testing the downstream property.

**Figure 4:**
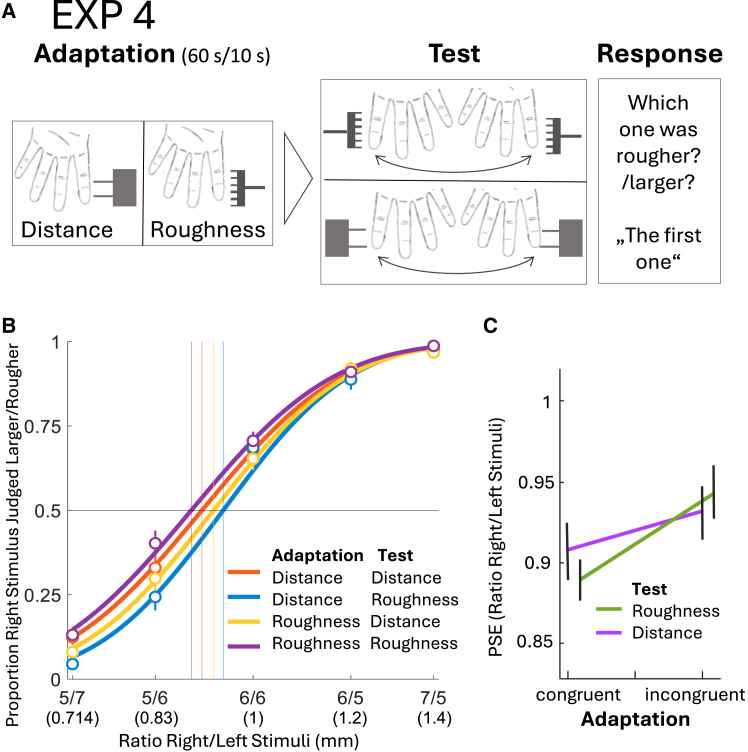
Procedure and results of experiment 4 (A) Experimental procedure, application area of the adaptation stimuli is always at the left-hand index finger pad, randomly applied across the entire surface. (B) Psychometric functions for the averaged proportions of the conditions distance adaptation/distance test, distance adaptation/roughness test, roughness adaptation/distance test, and roughness adaptation/roughness test (*N* = 26). Every data point shows the proportion of trials for which participants reported the test stimulus presented to the right index finger as rougher or larger than the stimulus presented to the left (adapted) index finger for each RIF/LIF stimulus ratio. Note, that participant’s actual response was whether the first or the second applied stimuli was rougher/larger. Psychometric functions are cumulative Gaussian functions. Error bars represent the SEM. Vertical lines represent mean PSEs of each condition. (C) Line plot depicting the average values of distance adaptation/distance test, distance adaptation/roughness test, roughness adaptation/distance test, and roughness adaptation/roughness test. Error bars represent the SEM.

In experiment 4, we aimed to infer whether a shared underlying mechanism drives the interactions between distance and roughness or whether the computation of one property might serve as initial upstream process, subsequently influencing perception of the other (Figure 3). To evaluate the potential (non-) hierarchical nature of the property-interactions, we tested and compared four aftereffect variants in the last experiment: two incongruent ones, i.e., the cross-property aftereffect from distance adaptation to roughness perception, and the reversed paradigm, i.e., roughness to distance; and the two congruent variants, i.e., roughness to roughness and distance to distance (Figure 4A). We were mostly interested in how the aftereffects differ between the incongruent and congruent conditions. If the properties rely on overlapping early neural computations before their representations diverge (common preprocessing), significant cross-property aftereffects are expected in both directions. However, cross-property aftereffects should be weaker than within-property aftereffects, since incongruent adaptation engages only part of the cues needed for computing each property. The greater the degree of common preprocessing, the smaller the difference between within- and cross-property aftereffects should be. If the properties share such early stages without an upstream-downstream relation, then cross-property aftereffects will be present but weaker than within-property aftereffects in both directions (see Figure 3A), which would manifest as a main effect of adaptation congruency in the ANOVA. By contrast, if the processing is hierarchically organized, cross-property aftereffects will also occur, but an additional asymmetry arises. Because processing of a downstream property necessarily begins with activation of the upstream property, adaptation to the downstream property stimulus will also engage the upstream stage (Figure 3B). Consequently, the difference between incongruent versus congruent adaptation would be null, or smaller when testing the upstream property as compared to the downstream property, because the upstream property would undergo intense processing even during incongruent adaptation, due to its early position in the processing stream. For instance, if distance is processed upstream to roughness, the magnitude of the aftereffects when testing distance should be similar regardless of whether distance (congruent) or roughness (incongruent) is adapted (i.e., smaller difference between incongruent and congruent adaptation). On the contrary, when testing roughness, congruent adaptation should produce larger aftereffects than adaptation to the upstream property (distance) (Figure 3B). This asymmetry between the two properties would manifest as an interaction between adaptation congruency and test property in the ANOVA. Results demonstrate that the cross-category aftereffect works bidirectionally, i.e., from tactile distance to roughness and vice versa, but for both test properties a congruent adaptation produces stronger aftereffects than an incongruent one, which might suggest early common shared processing of the properties instead of one property being an initial scaffold located upstream in the processing hierarchy.

A two-way repeated measures ANOVA with the factors congruency (congruent/incongruent) and test-property (distance/roughness) revealed a significant main effect of congruency (*F*[1,25] = 22.94, *p* = <0.001, *η*_p_^2^ = 0.48), with congruent conditions being associated with lower PSEs than incongruent conditions. There was no main effect of test-property (*F*[1,25] = 0.01, *p* = 0.94, *η*_*p*_^*2*^ = 0.01), and no interaction effect (*F*[1,25] = 2.55, *p* = 0.12, *η*_p_^2^ = 0.09). One sample *t* tests (one-sided, as aftereffects are expected for all conditions) revealed significant aftereffects for all conditions: congruent adaptation on distance test (distance to distance, *M* = 0.91, *SD* = 0.09, *t*[25] = −5.00, *p* = <0.001, *d* = −0.98), and roughness test (roughness to roughness, *M* = 0.89, *SD* = 0.07, *t*[25] = −8.08, *p* = <0.001, *d* = −1.59), as well as incongruent adaptation on distance test (roughness to distance, *M* = 0.93, *SD* = 0.07, *t*[25] = −4.70, *p* = <0.001, *d* = −0.92) and roughness test (distance to roughness, *M* = 0.94, *SD* = 0.08, *t*[25] = −3.85, *p* = <0.001, *d* = −0.76).

## Discussion

In this study we provide evidence that tactile distance, roughness, and curvature share early somatosensory processing by investigating if and under which circumstances perceptual adaptation aftereffects can transfer from one stimulus property to another. We observed cross-property adaptation-aftereffects where tactile distance adaptation influenced the subsequent perception of roughness. These effects arose from low-level cortical processing rather than from mere desensitization of peripheral receptors, as shown by the lack of adaptation aftereffects in control conditions using single-point stimuli applied randomly across the entire finger pad. In addition, we demonstrated bidirectional cross-property aftereffects, characterized by low-level features such as orientation- and location specificity. Notably, bidirectional aftereffects between roughness and tactile distance were weaker than the respective within-property aftereffects, suggesting that these tactile properties share early somatosensory processing stages rather than standing in a hierarchical relationship with each other.

Importantly, adaptation to distance affected not only roughness perception but also curvature perception, as evidenced in experiment 2 by contrasting aftereffects following adaptation to either a large or small two-point distance. This highlights the fundamental and generalizable nature of the observed effects: basic distance computation appears to follow similar initial processing as those mechanisms underlying the processing of spatial information from qualitatively distinct spatial inputs, which ultimately give rise to complex percepts such as curvature and roughness. Although our results do not establish neural-level causation, they demonstrate a functional causal link at the perceptual level: manipulating spatial encoding through adaptation systematically altered roughness or curvature perception. This change arises because adaptation temporarily modifies the responsiveness of the neural substrate encoding a certain feature.^11^^,^^36^^,^^37^ In summary, our findings demonstrated that computations which underlie somatosensory processing of different spatially characterized properties share common early mechanisms.

To evaluate the relationship between distance and roughness computation in detail, we compared cross-property aftereffects with their respective within-property aftereffects. The results suggest that, rather than one property serving as a foundational building block for the other, both properties may share a common early-stage preprocessing mechanism: for both properties, adapting to the same property (congruent adaptation) produced larger aftereffects than adapting to the other property (incongruent adaptation). If one property would be situated upstream in the somatosensory pathway as an initial step in the processing hierarchy of the other, then the difference between congruent and incongruent adaptation should be substantially smaller for that upstream-located property than for the other. As we found no evidence for this, one can speculate about other underlying mechanisms that might produce the observed cross-property interactions instead.

**Figure 5:**
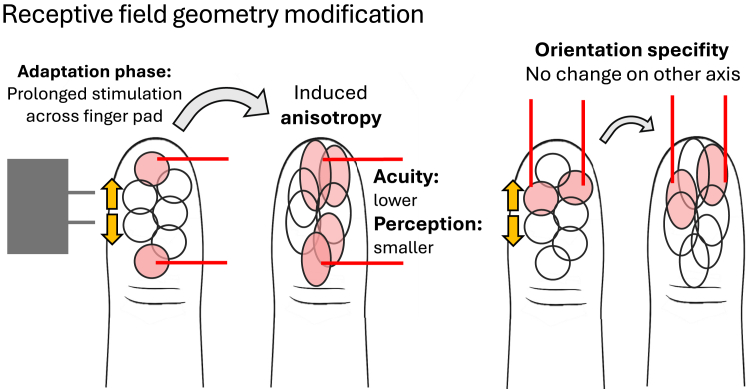
Schematic depiction of possible adaptation-induced modification of RF geometry at the finger pad Stimulation across finger pad with the distance-adapter might affect population-level receptive field organization across the stimulated region due to, e.g., intracortical inhibition release of neurons that represent the stimulated skin area. This would lead to reduced spatial resolution on one axis (where RFs then tend to be elongated and overlapping), but not the other.

Adaptation to large tactile distances may cause neurons specifically tuned to those distances to exhibit expanded RFs,^38^ reducing spatial resolution due to RF overlap.^39^^,^^40^ This would then lead to the perception of smaller distances^8^ or reduced roughness for subsequently presented smaller distances or finer gratings, due to the lower number of unstimulated RFs between the activation foci^8^ (see Figure 5). Such RF modulation may arise from lateral inhibition and plasticity at the network level, involving shifts or expansions in RF boundaries.^41^^,^^42^ The transfer of aftereffects between tactile properties suggests that the adaptation to the spatial property of distance is not strictly receptor-type specific.^10^^,^^43^^,^^44^ Rather, it may involve RF changes in neurons receiving convergent input from multiple afferent classes, contributing to all three properties due to their ability to encode both spatial details and sustained pressure.^10^ The larger aftereffects observed for congruent compared to incongruent adaptation may then be explained by later differences during higher-level property-specific processing downstream.^6^^,^^45^^,^^46^

There is evidence for correspondingly rapid RF-size modulation especially in the field of vision^47^^,^^48^ and also in touch.^38^^,^^49^ In the visual and the somatosensory system, lateral inhibition sharpens RFs by suppressing activity in neighboring neurons. For the tactile domain, pharmacological interventions have been shown to provoke a release from intracortical or thalamocortical inhibition, allowing excitatory regions of RFs to expand,^50^ while attentional manipulations have been found to boost inhibition and consequently decreased RF size.^49^ Similar mechanisms might account for the observed effects here, extending excitatory RF size through disinhibition in reaction to adaptation. This mechanism is particularly plausible for roughness perception, as supported by the spatial-variation model which proposes that roughness is determined via a computation of the variation of response patterns across sheets of RFs of SA1 afferents, with increased roughness perception for higher variation.^51^ If those RFs were to be enlarged, variation (and consequently the perceived roughness) across a certain area would decrease. A similar principle applies to curvature perception: as the RF enlarges, the perceived curved area decreases; leading to a higher perceived curvature.

The modulation of RF geometry as an underlying mechanism producing property interactions appears especially plausible given that the cross-property aftereffects observed in our experiments displayed low-level characteristics. Notably, the orientation specificity demonstrated in experiment 1 aligns well with the concept of RF modulation: if we assume that the RFs are stretched along one particular axis due to adaptation, e.g., the proximodistal axis, this deformation would result in decrease spatial information along that axis, reducing distance perception specifically in that direction. Consequently, no aftereffect should occur if the test stimulus is oriented orthogonal to the adapted axis, as RF geometry remains unaltered along that axis.^7^

Our findings provide robust evidence for the orientation specificity of the aftereffect from distance to roughness. Notably, prior studies have shown that stimuli are perceived as larger along the mediolateral axis of the finger than along the proximodistal axis, due to acuity anisotropies. This has been demonstrated in tasks involving grating orientation, two-point orientation, and gap discrimination tasks^52^^,^^53^^,^^54^ and the anisotropy is observed across different body parts.^1^^,^^55^ Consequently, if we had conducted the adaptation along the proximodistal axis and then applied test stimuli along the mediolateral axis to assess orientation-specificity, any observed lack of aftereffect could be mistakenly attributed to these anisotropies rather than true orientation specificity. Specifically, test stimuli that are physically smaller than the adapter may not be perceived as substantially smaller along the mediolateral axis due to higher acuity along that axis. This could obscure the expected aftereffect, which would normally cause the test stimuli to be perceived as smaller compared to stimuli applied on the non-adapted finger pad. To avoid this confound, we conducted adaptation along the mediolateral axis and tested along the orthogonal, proximodistal axis in experiment 1. This design ensured that any absence of the aftereffect could be attributed to orientation-specific mechanisms rather than perceptual anisotropies. Furthermore, in experiment 3 we applied both adapter and test on the mediolateral axis, which produced the typical aftereffect again. This reaffirms that the lack of aftereffect in experiment 1 was indeed due to rotation of the stimulus.

It is hence not likely that inherent RF geometry on the finger pad caused the lack of effect when the adaptation and test stimuli were applied on different axis. We therefore speculated that the orientation-specificity of the cross-property aftereffect between distance and roughness (experiment 1) might instead suggest involvement of orientation-selective neurons within the primary somatosensory cortex (SI), which are highly localized and finely tuned to specific orientations.^31^^,^^32^ Such neurons respond most robustly when stimuli are aligned with their preferred orientation, indicating that the neural mechanisms underlying the aftereffect are precise and spatially constrained.^31^^,^^32^ Given this premise, we did not expect the aftereffect to spread from the fingertip to the base of the finger. Although some propagation across adjacent skin regions within the same digit was anticipated due to cortical spread within SI,^34^ the absence of an effect on the middle phalanx in experiment 3 is consistent with previous findings demonstrating precise intra-digit maps within area 3b of SI, both in humans and macaques.^35^^,^^56^ Importantly, if the sensory mechanisms underlying the cross-property aftereffect involves geometry modulation of local RFs at early cortical processing stages, it is particularly plausible that the aftereffect would remain confined to the stimulated area without spreading to neighboring skin regions.

Previous studies have shown that coarse texture perception relies on spatial patterns of activation across mechanoreceptors, primarily slowly adapting SA1 afferents, and that spatial parameters such as inter-element spacing and groove width significantly influence perceived roughness.^23^^,^^24^^,^^25^ However, most of these studies inferred spatial coding either from stimulus-response mappings, where changes in tactile stimuli (like spacing or texture) are linked to perceptual outcomes without directly manipulating the underlying neural code,^2^^,^^20^ or from neurophysiological recordings, revealing correlations between neural firing patterns and specific spatial features.^3^^,^^21^ These methods suggest associations between spatial coding and perception but fall short of proving that directly altering the spatial representation itself, i.e., specifically, the metric of inter-point distance on the skin, leads to changes in tactile perception. Our study provides this causal evidence: modifying the spatial code through adaptation to a tactile distance systematically altered the perception of both roughness and curvature. Tactile distance adaptation primarily conveys spatial information by activating slowly adapting afferents (Merkel), while leaving rapidly adapting receptors (Meissner and Pacinian) mostly unaffected.^57^ Although our roughness test phases involved both spatial and temporal-vibratory information, since gratings were passively moved along the finger pad,^20^ the adaptation to two isolated points forming a specific distance still significantly influenced perception during the test phase. This highlights the critical role of slowly adapting afferents in computing macroscale roughness^21^^,^^22^ and demonstrates that spatial coding is not merely correlated with but is essential for the perception of coarse textures. It strengthens the Duplex theory by offering behavioral evidence that the spatial dimension of tactile input is a determining factor in roughness perception at the macro scale.

Experiment 2 demonstrated that adaptation to distance not only influenced roughness perception but also affected curvature perception. While both texture and distance share discrete spatial intervals as a common feature, curvature perception in passive touch is fundamentally different, as it arises from continuous deformation patterns on the skin when pressed against a surface. This difference makes the observed cross-property aftereffects between distance and curvature more surprising and intriguing. Notably, we found clear cross-property aftereffects between two-point distance and curvature for the large two-point stimulus. However, we did not observe differences between the two-point small distance adaptation and the single-point control, as both caused subsequently presented curvature stimuli to be perceived as less curved. One possible explanation for this result is that the single-point stimulus may have induced curvature adaptation on its own, potentially due to its perception as highly curved. Technically, each indentation of the distance stimulus produces a skin deformation similar to that caused by a small round stimulus, potentially resulting in a curvature percept to some extent.^5^ Under this premise, it is also possible that small distance adaptation had a similar impact as the single-point adaptation: although 1.5 cm is not below the average two-dot discrimination threshold,^58^ it might be the case that the small distance adapter was not consistently perceived as an actual distance, but rather as a single point. Previous studies on the tactile distance aftereffect also provided unclear results for small distance adaptation (2 cm), showing no substantial effects when a large distance was subsequently presented.^7^^,^^59^ This consistent lack of effects for small distance adaptation on the perception of larger distances makes it plausible that curvature adaptation might be responsible for the small distance adaptation on curvature perception observed here: if small distance adaptation does not influence large distance perception, it is plausible that it would also fail to affect the perception of larger length-areas. Nevertheless, the contrasting effect of the adaptation to a distance larger than the curvature stimulus provides strong evidence that tactile distance and curvature do share sensory processing. The large distance adapter did elicit the expected effect, increasing perceived curvature of subsequently presented stimuli.

In sum, our findings clearly demonstrate that the computation of different spatially defined tactile properties relies on a common initial processing step, with modulation of RF geometry emerging as a plausible candidate mechanism underlying the observed interactions. The observation that percepts derived from qualitatively different stimulus properties undergo the same primitive preprocessing is highly significant and provides a foundation for investigating broader principles in future works. Cross-property aftereffects effectively revealed shared somatosensory processing between properties within the current study, suggesting that, despite known distortions in somatosensory representations, early processing stages fundamentally encode spatial information. Employing such paradigms might hence be a useful tool in future studies to disentangle somatosensory computations and uncover previously unknown relationships.

### Limitations of the study

The study was conducted on participants within a limited age range (18–33 years) and test regions were exclusively located on the hands, which may limit the generalizability of the findings to other age cohorts or body sites. In addition, only one direction of the adaptation condition was tested in most experiments (using a larger adapter than the subsequently tested stimuli) for reasons of feasibility and parsimony. However, it could be of interest to examine whether the same effects are observed when using a smaller adapter. This consideration is particularly relevant because the expected effects were not observed with the smaller adapter in experiment 2, and other reports have similarly shown a lack of effect for smaller adapter stimuli.^7^^,^^59^

## Resource availability

### Lead contact

Requests for further information and resources should be directed to and will be fulfilled by the lead contact, Michaela Jeschke (michaela.jeschke@psychol.uni-giessen.de).

### Materials availability

This study did not generate new unique reagents.

### Data and code availability

Raw individual data (MATLAB files) and derived PSE values (csv files) have been deposited at zenodo and are publicly available as of the date of publication at https://doi.org/10.5281/zenodo.14809561. All original analysis code has been deposited at zenodo and is publicly available at https://doi.org/10.5281/zenodo.17314317 as of the date of publication. Any additional information required to reanalyze the data reported in this article is available from the lead contact upon request.

## Acknowledgments

We want to thank Hannah Wilkening, Marai Söhngen, Kimberly Glas, Lars Leon Hagemeier, and Jessica (who has no surname) for their help with running the experiments. Research was supported by the 10.13039/501100001659Deutsche Forschungsgemeinschaft (DFG, German Research Foundation) – project no. 222641018 – SFB/TRR 135, A5.

## Author contributions

M.J. designed the research, performed experiments, analyzed data, interpreted results of experiments, prepared figures, drafted manuscript, edited and revised manuscript, and approved of the final manuscript. E.A. designed the research, interpreted results of experiments, edited and revised manuscript, and approved of the final manuscript. K.D. designed the research, interpreted results of experiments, edited and revised manuscript, and approved of the final manuscript.

## Declaration of interests

The authors do not declare any competing interests.

## STAR★Methods

### Key resources table

**Table:** 

REAGENT or RESOURCE	SOURCE	IDENTIFIER
**Deposited data**		

Data (behavioral)	Zenodo repository	https://doi.org/10.5281/zenodo.14809561

**Software and algorithms**		

Custom analysis code	Zenodo repository	https://doi.org/10.5281/zenodo.17314317
MATLAB Version 2022b	Mathworks	R2022b, https://it.mathworks.com/products/matlab.html
Palamedes Toolbox	Prins, N. & Kingdom, F.A.A. (2018)^60^	https://www.palamedestoolbox.org/download.html
JASP Version 0.18.3	JASP Team (2024)	https://jasp-stats.org/

### Experimental model and study participant details

For experiment 1, due to the very large effect sizes reported for the tactile distance aftereffect (*d*_*z*_ ≥ 1.59),^7^ we expected a medium-to large effect (*d*_*z*_ = 0.65). Based on that, we conducted an *a priori* sample size calculation for a power of 80% and an alpha of 5%. Projected sample size was 17 for the one sided one-sample *t* test and 17 for a matched-pairs two sample *t* test (G∗Power^61^). To allow for complete Latin-square randomization of experimental conditions we collected data from 18 right-handed students from Justus-Liebig University Giessen (14 female, age 18–33 years, mean: 23.29 years). For experiment 2, based on the results of experiment 1, we expected large effects for a transfer of the tactile distance aftereffect to curvature perception and consequently collected data of 18 participants, following similar power-considerations (11 female, age 18–30; mean: 22.38). For experiment 3, we again based the power analysis on the expectation of a medium-to-large effect (*d* = 0.65) for an aftereffect on the middle phalanx, yielding the sample size of 17 with a power of 80% for one-sided one sample test and a one-sided matched pairs *t* test (alpha 5%). Based on these power considerations, we collected data of 18 right-handed students (11 female, age 19–29 years, mean: 23.78 years). For experiment 4, based on the large effect size (*d* = ca. 1) observed for the cross-property effect on the finger pad in Exp. 1/3, we expected a medium-sized (*d* = 0.5) congruency effect (i.e., stronger PSE-shift for congruent adaptation than for incongruent) when the tested property is roughness. Under the assumption of a hierarchical processing model, we would not expect such a congruency effect when distance is the tested property, resulting in a medium sized (*d* = 0.5) two-by-two interaction effect in a repeated measures ANOVA (power 0.8), yielding a required sample size of *N* = 27. We collected data from 26 participants (13 female, age 18–30 (mean 24.0).

None of the participants reported cutaneous impairments or sensory deficits. We confirmed that by conducting a two-point discrimination test; all had two-point discrimination thresholds lower than 3 mm on their index fingers.^62^ They were recruited in the city of Giessen, Germany; with middle socioeconomic status. Most of them have European origin. The experiments employed within-subjects designs, i.e., there were no experimental groups, which controls for between-subjects effects due to sex or gender. All participants were naive to the purpose of the experiment, provided written informed consent, and received financial compensation (8€/hour). The experiment was approved by the local ethics committee LEK FB06 and conducted in accordance with 2013 Declaration of Helsinki, except for preregistration. None of the participants had participated in the previous experiments, respectively.

### Method details

#### Experiment 1

##### Stimuli and setup

Participants sat in front of the experimenter with their hands lying next to each other on a hand rest (height 10 cm, distance between finger pads of the index fingers approx. 25 cm), palms facing upwards, blindfolded, and wearing headphones with active noise-cancelling. A computer with MATLAB R2020b (MathWorks Inc. 2007) was used to guide the experimenter and collect responses. The experimental procedure was built using the Psychophysics Toolbox.^63^ All haptic stimuli were 3D-printed (Figure 1A; printer: Formlabs Form 3, XY-resolution of 25 microns, layer thickness of 25–300 microns). We used two different adaptation stimuli consisting of either one spike (single-point adaptation stimulus) or two spikes (two-point adaptation stimulus) separated by 4 mm (between their inner edges). With the intention of using similar test stimulus ratios as in Calzolari et al.,^7^ we selected the adaptation distance value of 4 mm after consideration of natural constraints in both directions imposed by finger pad size and tactile acuity limits.^62^ For similar reasons, we did not include an additional adaptation condition with a smaller distance (e.g., 2 mm), as this would not have been feasible given acuity constraints.^62^ Notably, several experiments in Calzolari et al.^7^ also omitted a smaller adaptation condition for reasons of parsimony. Each spike had a radius of 1 mm at their tip and a height of 8 mm and were placed on top of a 25 mm × 20 mm × 20 mm cube. Test stimuli were gratings with a groove width of either 2-, 3- or 4-mm. Ridge width was 1 mm and amplitude was 3 mm for all gratings. The gratings were 3D-printed with a surface of 25 mm × 20 mm × 5 mm and a handle (height: 30 mm, diameter: 5 mm) at their bottom. We chose an amplitude size that prevented ground contact to maintain the linear relationship of element spacing and perceived roughness.^23^

##### Procedure

Each trial consisted of an adaptation phase and a test phase. During the adaptation phase, blindfolded participants were touched across the left index finger pad with either the two-point adaptor along the proximodistal axis, i.e., across the length of the finger pad (same orientation condition), along the mediolateral axis, i.e., across the width (orthogonal rotation condition) or with the one-point adaptor (single-point orientation condition), in three separate conditions (order counterbalanced across participants using a Latin square design). The application area was defined as depicted in Figure 1A, and individual applications of the adaptation stimulus were evenly distributed across the area, with distinct space between consecutive applications, so that the stimulus was never applied systematically to the exact same points on the skin. The aim was to adapt to the abstract property of distance (i.e., the spatial relation between two tactile events), rather than to adapt two exact locations on the skin. The single-point control stimulus was applied in the same way; with applications distributed across the area. The adaptation was conducted for 10 s before each trial except for the first trial after each break and at the start of each condition, for which we implemented an intensive 60 s adaptation phase. Adaptation durations were the same as those used by Calzolari and colleagues.^7^ After the 10 (or 60) secs adaptation, two test stimuli were applied sequentially, one to each index finger pad. The test stimuli consisted of gratings of 2-, 3-, and 4-mm groove width presented in 5 possible combinations: right index finger/left index finger (RIF/LIF): 2/4, 2/3, 3/3, 3/2, 2/4, forming the RIF/LIF ratios 0.5, 0.66, 1, 1.5 and 2. These ratios are equivalent to the ones used by Calzolari and colleagues.^7^ Note that the right index finger is used here as a comparison area which should not be affected by adaptation to the left finger.^7^ Test stimuli were applied with a continuous stroking movement in the proximodistal direction as depicted in Figure 1B, with the ridges being oriented horizontally from the participants’ perspective. To eliminate contrast enhancement due to simultaneous stimulation, discrimination performance was assessed in a sequential two-interval forced choice (2IFC) task. Application of test stimuli started randomly and equally often on the left and the right hand and participants had to judge if they perceived the first or the second stimulus to be rougher, responding verbally with no time restriction. The total trial number was 180 (5 pairs (ratios) x 3 conditions × 12 repetitions per pair). Adaptation as well as test trials were conducted using passive touch, eliminating motor and proprioceptive feedback and the possibility to actively alter scanning speed and pressure in order to gain additional information. Importantly, the experimenter had undergone a training beforehand to replicate the pressure and timing between touches that experimenters used in the studies on tactile distance aftereffects by Calzolari and colleagues.^7^ Nonetheless, we do not suspect pressure or timing of stimulation to be highly relevant in the context of producing these aftereffects. The experiment took ∼1.5 h, with 10-min breaks between the three main blocks that constituted the three adaptation conditions.

##### Quantification and statistical analysis

Behavioral data of *N* = 18 human individual participants was collected and analyzed. All statistical analyses were performed using the MATLAB (Mathworks Inc.) and JASP software. The analyses performed can be found in the subsection of the results section: experiment 1. For each participant, we first computed the proportion of trials in which they judged the RIF stimulus to be rougher; separately for each RIF/LIF ratio and each adaptation condition. For statistical analyses and fitting the data, we used common logarithms of the five RIF/LIF ratios to produce a symmetrical distribution from the point of actual equality (x = 1). For intuitive interpretation, we converted the mean of the logarithms back into ratios. For each of the three adaptation conditions separately, we fitted cumulative Gaussian distributions (two free parameters: μ [alpha] and σ [beta]) as functions of the logarithmic RIF/LIF ratios to the individual participants’ data, using a Maximum Likelihood criterion. For that, we used the Palamedes Toolbox implemented in Matlab^60^ The points of subjective equality (PSEs) were defined as the estimated RIF/LIF stimulus ratios at which participants were equally likely to judge either the LIF or the RIF stimulus as rougher. As an indicator of the perceptual bias, we use the difference between the PSE and the point of objective equality = 1. PSE values smaller than 1 indicate a tendency to perceive a texture applied to the adapted finger as less rough than without adaptation. PSE values larger than 1 indicate the opposite. Due to a violation of the assumption of normality (*p* = <0.001 in a Shapiro-Wilk-test), we conducted a Friedman test on the individual log PSEs with the within-participant factor adaptation condition (same, orthogonal, single-point) in combination with Conover’s post hoc tests for all adequate comparisons. Additionally, we conducted Wilcoxon rank-tests against log 1 (=zero) for the median log PSE values of each of the three adaptation conditions. Consequentially, we reported median and median absolute deviation in the results subsection. The significance level was set at 5% in all analyses for all experiments. Whenever we had a clear hypothesis on the expected direction of an effect, we employed one-sided t-tests or their non-parametric equivalents. This is explicitly stated throughout the results subsection of all experiments. Otherwise, two-sided tests were used, which is not explicitly stated. Further, all post-hoc tests were conventionally conducted as two-sided. Average psychometric functions of all conditions are depicted and explained in Figure 1C and the respective figure caption. Single data points are depicted in Figure 1D and explained in the respective figure caption.

#### Experiment 2

##### Stimuli and procedure

To assess cross-property aftereffects, participants' left-hand palm was adapted before each trial either to a distance stimulus (1.5 cm) that is smaller than the indentation area of a subsequently presented curvature stimulus, to a larger stimulus (4.5 cm), or the control single-point stimulus. Afterward, a 2IFC discrimination task followed, where they had to report which passively applied curvature stimulus was more curved (see Figure 2A). All stimuli were 3D-printed curvature models (Figure 2B, printer: Ultimaker S5, XY-resolution of 6.9 microns, layer thickness of 20–200 microns), constructed after thorough prototyping that allowed ergonomic application at the palm and adequate difficulty levels similar to Exp.1. We ensured that while indenting, the whole length of the curve was in contact with the skin, and not only (varying) parts of it. The final stimuli selected included three cylinder-segments with a constant width of 2 cm and length of 4 cm, placed on top of a 2 × 4 × 1 cm base (see Figure 2B). Differences in curvature between the cylinder segments are therefore only reflected by height differences between the segments, with base-to-peak heights of 0.536, 0.376, and 0.271 cm. By keeping segment length constant, which would otherwise vary proportionally with curvature, we avoid confounds that could complicate later interpretations.^9^ The segments were part of cylinders with radii of 4 cm, 5.5 cm, and 7.5 cm and resulting curvature values (conventionally expressed as R^−1^) of 0.25, 0.1818, 0.1333 cm^−1^. The large-distance stimulus comprised a two-point distance of 4.5 cm and the small-distance stimulus comprised 1.5 cm (which is feasible as the two-point discrimination threshold at the palm is 0.75 cm^58^). The single-point stimulus again served as control. Following the same logic as the previous experiment, test stimuli were presented in 5 possible combinations: right hand/left hand (RH/LH): 0.13/0.25, 0.13/0.18, 0.18/0.18, 0.18/0.13, 0.25/0.13, forming the RH/LH ratios 0.53, 0.73, 1, 1.36, 1.86. The total trial number was 210 (5 pairs (ratios) × 3 conditions × 14 repetitions per pair). Test procedure was similar as in Exp. 1 except that the application area here was at the palm, to allow for feasible discrimination performances and stimulus construction. Distance adaptation stimuli were always presented in proximodistal orientation (along the length of the hand) and test stimuli were statically pressed onto the skin for ca. 1 s (along the length of the hand). The experiment took ∼1.75 h, with 10-min breaks between each of the three main blocks and a 2-min break halfway through each main block.

##### Quantification and statistical analysis

Behavioral data of *N* = 18 human individual participants was collected and analyzed. Preprocessing of data and calculation of PSE-values was similar to Exp. 1. We performed a repeated-measures ANOVA on log PSEs with the within-participant adaptation type (levels: small, large, single-point) and t-tests against log 1 (=zero) for the mean log PSE values of each of the three adaptation conditions. The analyses performed can be found in the subsection of the results section: experiment 2. Further, we reported means with standard deviations in the results subsection. Average psychometric functions of all conditions are depicted and explained in Figure 2C and the respective figure caption. Single data points are depicted in Figure 2D and explained the respective figure caption.

#### Experiment 3

##### Stimuli and procedure

On each trial, participants were first adapted to a distance stimulus across their left finger pad. Again, a 2IFC-discrimination task followed, where gratings were either applied across their finger pad, across the middle phalanx, or across the base of the finger (see depiction in Figure 1E), and they had to report which one they perceived to be rougher. Same stimuli were used as in Exp. 1: gratings of 2, 3, 4 mm groove widths and a 4 mm distance stimulus as adaptation stimulus. Application axis of adaptor and test stimuli was always mediolateral (i.e., only one adaptation condition). Test trials were conducted in random order at three possible areas: finger pad (same as the adapted area), middle, base of the finger. Total trial number was 180 (5 pairs (ratios) × 3 test area conditions × 12 repetitions per pair). The experiment took ∼1.5 h, with 10-min breaks between each of the three main blocks and a 2-min break halfway through each main block.

##### Quantification and statistical analysis

Behavioral data of *N* = 18 human individual participants was collected and analyzed. We performed a repeated-measures ANOVA on log PSEs with the within-participant factor “test area” and conducted one-sample t-tests against zero for the mean log PSE values of each of the three testing area conditions. The analyses performed can be found in the subsection of the results section: experiment 3. We again reported means with standard deviations in the results subsection. Average psychometric functions of all conditions are depicted and explained in Figure 1E and the respective figure caption. Single data points are depicted in Figure 1F and explained in the respective figure caption.

#### Experiment 4

##### Stimuli and procedure

On each trial, first participants underwent an adaptation phase at the finger pad to either roughness or distance, depending on the condition. Afterward, a test phase followed, either with distance stimuli or grating stimuli (see Procedure Figure 3A). To ensure adequate discrimination performance, we used tactile distances of 5, 6, 7 mm and in compliance with that, gratings with groove widths of 5, 6, 7 mm, forming the RIF/LIF ratios 5/7 (0.714), 5/6 (0.83), 5/5 (1), 6/5 (1.2), and 7/5 (1.4). Distance and grating stimuli of 7 mm (distance or groove width, respectively) served as adaptation stimuli, depending on the condition. The experimental design comprised the two factors “congruency” (adaptation condition) and “test property”. Test property was either distance or roughness. In the “congruent” adaptation condition, the adapted property matched the property tested afterward. In the “incongruent” condition, a different property was adapted than the one subsequently tested. The experiment consisted of four blocks; throughout the first two blocks, either distance or roughness was adapted. Throughout the latter two blocks, the other property was adapted, respectively. Order of adapted property (distance first/roughness first) was counterbalanced between participants. Within each block, trial order was randomized and the test property hence could vary from one trial to another. Total trial number was 240 (12 repetitions per ratio (5) × adaptation condition (congruent/incongruent) × test condition (distance/roughness). Adaptation and test area were at the left index finger pads, and adaptation as well as test application axis was proximodistal. The experiment took ∼2 h, with 10-min breaks between each of the four main blocks and a 2-min break halfway through each main block.

##### Quantification and statistical analysis

Behavioral data of *N* = 26 human individual participants was collected and analyzed. We performed a repeated-measures ANOVA on log PSEs with the within-participant factors “Congruency” and “Test property” and four one sample t-tests. The analyses performed can be found in the subsection of the results section: Experiment 4. We again reported means with standard deviations in the results subsection. Average psychometric functions of all conditions are depicted and explained in Figure 4B and the respective figure caption. A line plot depicting the average PSE values and the standard error of the mean of all conditions is depicted and explained in Figure 4C and the respective figure caption.
